# Combining Enhanced Resolving Power with Duty Cycle Improvements on a Multi-Reflecting Time-of-Flight Mass Spectrometer

**DOI:** 10.1021/jasms.4c00122

**Published:** 2024-08-09

**Authors:** William J. Johnson, Martin E. Palmer, Emmanuelle Claude, Michael McCullagh, Peter Nixon, Jason Wildgoose

**Affiliations:** † Waters Corporation, Stamford Avenue, Altrincham Road, Wilmslow, Cheshire SK9 4AX, U.K.

## Abstract

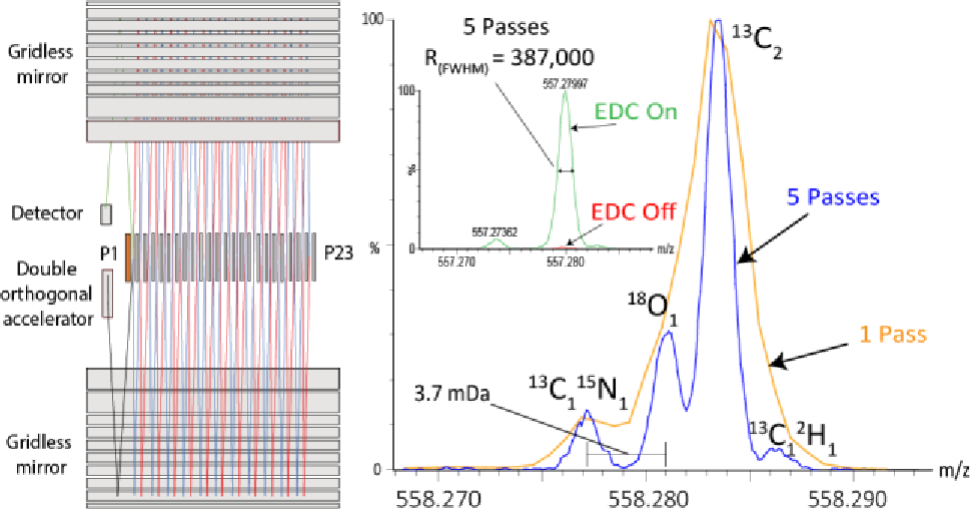

The combination of enhanced resolving power and improved duty cycle on a multireflecting time-of-flight mass spectrometer is described. Resolving power increases are achieved by extending the effective ion path length from 47 m to greater than 200 m. Path length increases are achieved through containment of ions within the analyzer for up to *N* = 5 passes using a pulsed deflection electrode. Resolving power was shown to increase from 220,000 to 402,000 (fwhm) at *m*/*z* 785 for *N* = 1 and *N* = 4 analyzer passes, respectively. Due to the timing of the pulsed deflection electrode, the approach is particularly suited to high resolution analysis over a targeted *m*/*z* range. Duty cycle enhancements are achieved for ions of the targeted *m*/*z* range via accumulation prior to orthogonal acceleration, providing signal improvements of 2 orders of magnitude. Achieving such high resolving powers at fast scan rates (30 Hz) can yield additional information such as fine isotope structure; when combined with ppb mass measurement accuracy, high confidence in analyte identification can be achieved. The technique is applied for *N* = 2 analyzer passes, demonstrating fine isotope structure for a typical UHPLC metabolite identification experiment at a 10 Hz acquisition rate. Additionally, mass spectrometry imaging data is acquired using DESI, demonstrating the improved image clarity achieved at >300,000 (fwhm).

## Introduction

The introduction of multireflecting time-of-flight (ToF) mass analyzers has enabled exceptional levels of *m*/*z* resolving power.^1,2^ Minimization of high-order flight time aberrations have led to *m*/*z* resolving powers in excess of 200,000 fwhm (*m*/*z* 785) for ion path lengths of 47 m, while minimal ion losses within the analyzer are achieved due to grid-less ion mirrors.^3^ Further improvements in the resolving power of ToF mass analyzers are particularly advantageous due to the speed and high mass accuracies inherent to the technique. Since resolving power is proportional to the ion flight time *T*, typically in the order of milliseconds, high *m*/*z* resolving power may be achieved over extremely short time periods. ToF systems are therefore very suited to fast upstream separation techniques and experiments where speed is advantageous, such as UHPLC MS and MS imaging.

Further improvements to instrumentation yielding resolving powers >300,000 fwhm at fast scan speeds of up to 30 Hz can provide additional information for characterization.^4^ Isolating *m*/*z* interferences or fine isotope structure provides further confidence in analyte identification and can significantly reduce the number of possible identifications. For MS imaging applications, higher resolving powers can also provide further detail on spatially localized analytes of interest, significantly improving image clarity. A particular benefit of the long stable flight times on the multireflecting time-of-flight system is high mass measurement accuracy, typically less than 200 ppb. This can significantly reduce the number of possible chemical formulas assigned to a peak.^5^ For analytes with *m*/*z* < 700, a mass accuracy of 200 ppb significantly reduces the number of possible formulas, enabling more confident identifications. For analytes of *m*/*z* > 700, additional information may be required to confidently identify the analyte of interest. Fine isotope structure can be used for identification since this can rule out other possible chemical formulas. In order to resolve fine isotope structures, further improvements in *m*/*z* resolving power can be advantageous. For time-of-flight mass spectrometers, resolving power can be defined as
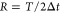
1where Δ*t =* Δ*t*_0_ + Δ*t*_aber_ is the ion arrival time distribution at the detection plane.^6,7^ Extending the time-of-flight *T* or decreasing Δ*t* will lead to increased resolving power. The flight time aberrations Δ*t*_aber_ define the packet expansion due to the ion mirror system. These have been described previously on a hybrid quadrupole-multireflecting time-of-flight mass spectrometer and demonstrated up to fourth-order chromatic focusing,^2^ Δ*t*_0_ is limited by the formation of the beam prior to orthogonal acceleration and the high field during acceleration, typically in the order of nanoseconds. An additional time limitation can be attributed to the detection system; the response time (fwhm) for a single ion event after amplification can typically range from 500 ps up to 1.5 ns. While all these factors can be addressed to reduce Δ*t*, improvements in detection systems, phase space during orthogonal acceleration, and ToF aberrations within the analyzer itself can be challenging to achieve. Increasing *T* for the system described here has therefore been explored in detail.

The multireflecting time-of-flight system described previously can be characterized as an open-loop system.^3^ The advantage of such a system is a theoretically unlimited mass range, since overlapping of varying ion *m*/*z* does not occur over a fixed path length from the orthogonal accelerator to the detector. Increasing *T* can be achieved by either physically increasing the dimensions of the analyzer or by restricting ions for multiple passes; there are clear advantages to being able to increase path length within a constant geometry. Creating a closed-loop flight path in order to contain ions for multiple analyzer passes has been extensively demonstrated to result in high resolving powers for a targeted *m*/*z* range.^1,8−10^ The multireflecting ToF analyzer described here utilizes a pulsed potential difference applied to the first periodic lens *P1* to restrict the path of ions and form a closed-loop. To prevent overlapping of low *m*/*z* ions with higher *m*/*z,* restrictions on the duration of the *P1* pulse are introduced. The approach is therefore advantageous for improving resolving power of a targeted *m*/*z* range and has been demonstrated to achieve in excess of 500,000 (fwhm) for five analyzer passes at *m*/*z* 502.^1^ However, due to the proportional increases in flight times (several milliseconds) with additional number of passes, duty cycle can be significantly reduced.^11^ Methods for recovering duty cycle coupled with multiple analyzer passes are therefore advantageous. Accumulating ions prior to orthogonal acceleration has been shown to significantly enhance duty cycle (EDC) on quadrupole time-of-flight mass spectrometers.^12^ Multiple analyzer passes combined with ion accumulation prior to orthogonal acceleration are described here in detail. Resolving power increases of up to 402,000 (fwhm) for four analyzer passes and duty cycle improvements of 2 orders of magnitude are observed. The technique is applied to a typical UHPLC metabolite identification experiment as well as demonstration of improvements in image clarity for DESI mass spectrometry imaging.

## Instrument Design

### Multiple Passes of the MRT

**Figure 1:**
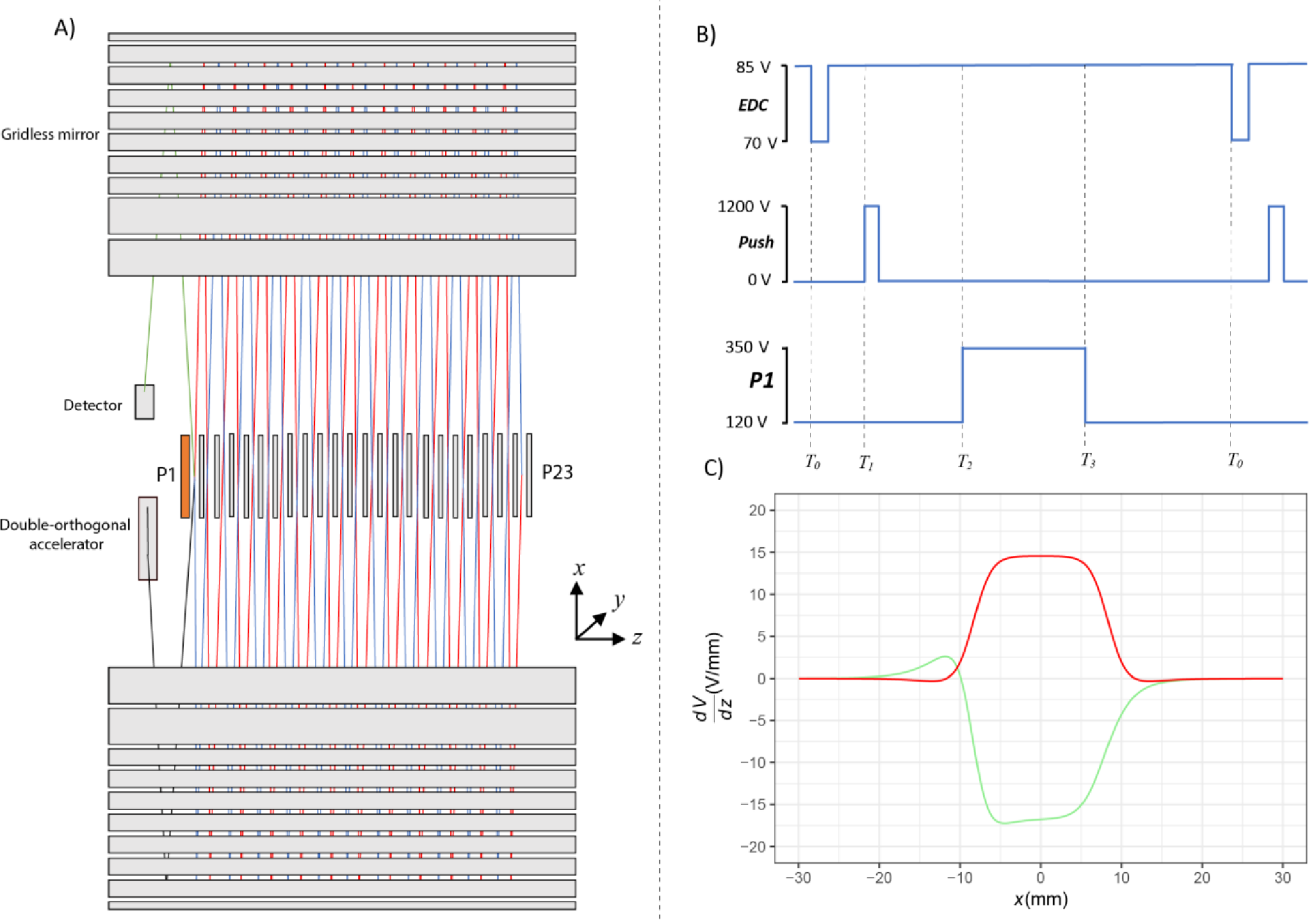
(A) Schematic of the MRT analyzer showing ion path for multiple passes. Ions enter *P1* from the orthogonal accelerator (black), where they are deflected into the periodic lenses. The beam follows a “zig-zag” path (red) until it reaches *P23*, where the ions are deflected back in the opposite *z*-direction (blue). When ions reach *P1* again, the ion path can either be deflected to the detector (green) or the potential may be adjusted such that the beam follows another pass of the analyzer (red). (B) Timing diagram of pulse timing for EDC, push, and *P1* deflection. Accumulated ions are released from the collision cell at *T*_0_ and arrive at the orthogonal accelerator with an *m*/*z* dependent time delay *T*_1_ – *T*_0_. Ions pushed from the orthogonal accelerator at *T*_1_ travel into the grid-less mirror assembly and subsequently through *P1*. At time *T*_2_, a pulse is applied to *P1*, creating a closed loop to generate multiple passes within the analyzer. The start time of the pulse *T*_1_ can be used to define the lowest *m*/*z* to make an additional pass of the analyzer, and the duration of the pulse *T*_3_ – *T*_2_ defines the upper *m*/*z* range and the number of analyzer passes ions make. (C) Modeled field strength d*V*/d*z* using a reference ion of mean energy 6 keV along the *x*-axis within the *P1* lens with the pulse applied (red) to generate another pass or not applied (green) for the ion to be deflected to the detector. The asymmetry of the field in the green series is a consequence of the larger deflection angle (∼1.6°) required to reach the detector, and the reference ion travels a path closer to the edge of the *P1* aperture where fringe field effects can be increased.

In the multireflecting time-of-flight analyzer described, ions are transmitted into the grid-less ion mirrors via an orthogonal accelerator; they are then reflected into a periodic lens and transmitted to an opposing ion mirror (Figure 1A). The periodic lens ensures divergence of the ion beam in the *z*-axis (typically <2 mm) is minimized; this improves both ion transmission and reduces the spread of ion arrival times.^13^ The reflection of ions between the mirrors and the periodic lens is repeated 23 times before a deflection lens *P23* pushes the ions back in the opposite *z*-direction toward the detector. The path is repeated back to the first periodic lens *P1*. The *P1* aperture is split, such that an independent deflection potential can be applied to steer the ions in the *z*-axis either to the detector (±120 V) or for another pass of the analyzer (±350 V) (Figure 1C). The green series illustrates the change in field for a reference ion path to the detector; this is approximately 1° greater than the path deflected back to the periodic lens for another pass (red), resulting in the field asymmetry observed. The effects on ion arrival time distribution have been explored in detail, including methods for decreasing time front tilts using local wedge electrodes.^13^ On the system described here, the beam width in the *z*-axis is <1 mm and approximately 6 times smaller than the aperture width of the periodic lens, minimizing the effect on ion arrival time distributions. For multiple passes, the pulsed potential on *P1* may be tuned such that an optimum trajectory into subsequent periodic lenses is achieved; since the beam dimension in the *z*-axis (<1 mm) is much smaller than the periodic lens aperture (6 mm), maximum ion transmission through the analyzer is achieved. For multiple analyzer passes, the timing of the pulse on *P1* is applied such that ions of a given *m*/*z* arriving at *P1* are deflected back for another pass of the analyzer. The start time of the pulse (rise time of <100 ns) is carefully matched to the lowest *m*/*z* of interest, and the duration defines the upper *m*/*z* range and the number of analyzer passes. Using this approach, the ion path length can be extended to greater than 200 m within a 0.5 m^2^ analyzer.

### Path Length Increases for Multiple Analyzer Passes

Path length increases and relative ion arrival times for additional analyzer passes on the system described above are defined as the summations of the red and blue trajectories shown in Figure 1. The total ion path length increase *A* for *M* additional passes of the analyzer is given by
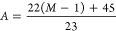
2

For the *M* = *N* + 1 additional analyzer pass, the path length is slightly less than double, *A* = 1.96, since the path from the orthogonal accelerator to *P1* (black) and *P1* to the detector (green) is only completed for ions entering the analyzer or ejected to the detector. A path length increase to 227 m (*A* = 4.83) is achieved for five analyzer passes; for an ideal system using eq 1, a similar proportional increase in theoretical resolving power may be observed. In practice, flight time aberrations accumulate over multiple passes and provide a broadening of the arrival time distribution 2Δ*t* for longer path lengths; this can significantly limit the improvements in achievable resolving power.

### *m*/*z* Ranges for Multiple Passes

Due to the timing of the pulse on *P1*, only a limited *m*/*z* range may be deflected for another pass in the analyzer. As the number of passes increases, the *m*/*z* range available decreases before overlapping of low *m*/*z* ions with higher *m*/*z* ions occurs. The rising edge of the push pulse in the OA is defined as *T*_1_ (Figure 1B). The start time of *P1* pulse *T*_2_ defines the lowest *m*/*z* that is contained for a second pass, and the end of the pulse *T*_3_ defines the largest *m*/*z*. However, to ensure that only one additional pass occurs, *T*_3_ must be less than the time taken for the lowest *m*/*z* to pass the analyzer twice.

The number of passes through each periodic lens can be used to evaluate the *m*_max_/*m*_min_ ratio for the *N* analyzer passes. In general, this is defined as
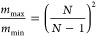
3

However, to account for the ion path length from the orthogonal accelerator to *P1* and to the detector, the ratio can be described in more detail. For two passes of the analyzer, the lowest *m*/*z* must have made no more than 44 passes of the periodic lens system. The *P1* deflection potential must then stay high for a duration such that the highest *m*/*z* has made at least 45 passes of the periodic lens in order to arrive at *P1* and not be deflected for another analyzer pass. To ensure overlapping of ions does not occur, the *m*/*z* range of interest can be characterized as
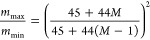
4

Following the constraints in eq 4, for two analyzer passes the *m*_max_ = 1564 with a corresponding *m*_min_ of 400, while for five analyzer passes *m*_max_ = 623. For an increasing number of passes, the technique is suited to analysis of narrower *m*/*z* ranges.

### Enhanced Duty Cycle (EDC)

As flight times are extended for multiple analyzer passes, a decrease in duty cycle is observed. Duty cycle *D* can be defined as the ratio of the fill time *t*_fill_ within the orthogonal accelerator to the total flight time of ions *T* of a given *m*/*z*.^11^
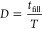
5

On the system described, ion flight times *T* can be on the order of milliseconds, while fill times are typically on the order of microseconds.

**Figure 2:**
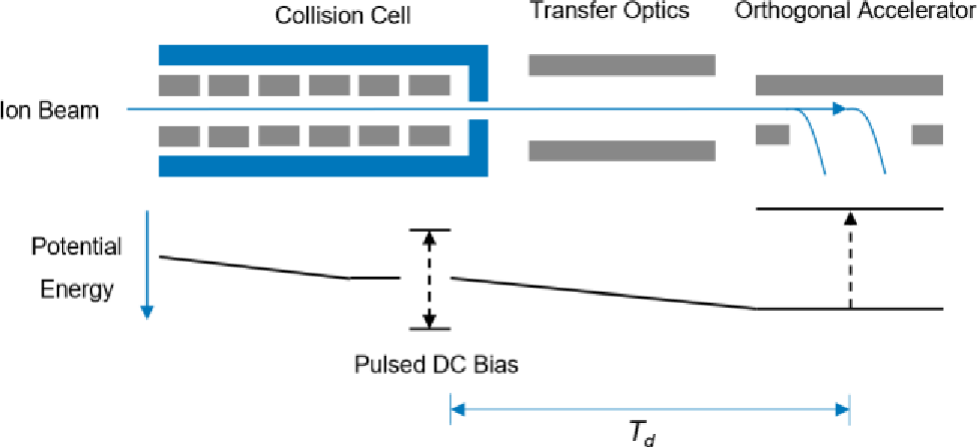
Ions are accumulated in the collision cell using a pulsed DC bias; when the bias is reduced, ions are released and arrive at the orthogonal accelerator after a time *T*_d_ = *T*_1_ – *T*_0_ (typically in the order of tens of microseconds) dependent on their *m*/*z*. Synchronizing the push at the orthogonal accelerator for a desired *m*/*z* can greatly increase effective duty cycle.

Without using any methods for enhancing duty cycle, for a single pass of the analyzer, the system duty cycle is approximately 0.1% and for two passes approximately 0.05%. Clearly, without a method of increasing duty cycle, multiple analyzer passes would suffer from an increasing reduction in sensitivity, which would be impractical for most applications. For single passes of the analyzer, approaches to recover duty cycle have previously been discussed.^3,11,12,14−16^ One such approach uses encoded frequent pushing,^11^ where multiple pushes are performed within a discrete time interval; the time differences between pushes are unique, enabling different ion arrival times for the same *m*/*z* to be subsequently decoded into a coherent spectrum. This has been shown to achieve up to 2 orders of magnitude in signal recovery per scan when compared to a conventional “push and wait” approach. However, this approach is not simple to apply when deflecting ions within the analyzer for multiple passes as multiple pushes can cause ions of a given *m*/*z* to arrive at *P1* at differing times. This can cause challenges in decoding the resultant multiplexed spectra. Another approach to significantly improve duty cycle is to accumulate ions prior to orthogonal acceleration (Figure 2).^12^ Using a pulsed potential well in a collision cell upstream of the orthogonal accelerator, ions accumulate during the period of the previous push. Ions are released with a delay to the orthogonal accelerator such that the *m*/*z* range of interest arrives at the time of the push. Since ions are bunched using this enhanced duty cycle (EDC) approach and subsequent ions are stored for the next push, depending on the trapping efficiency and ion optics to the orthogonal accelerator, significant duty cycle improvements can be made.

**Figure 3:**
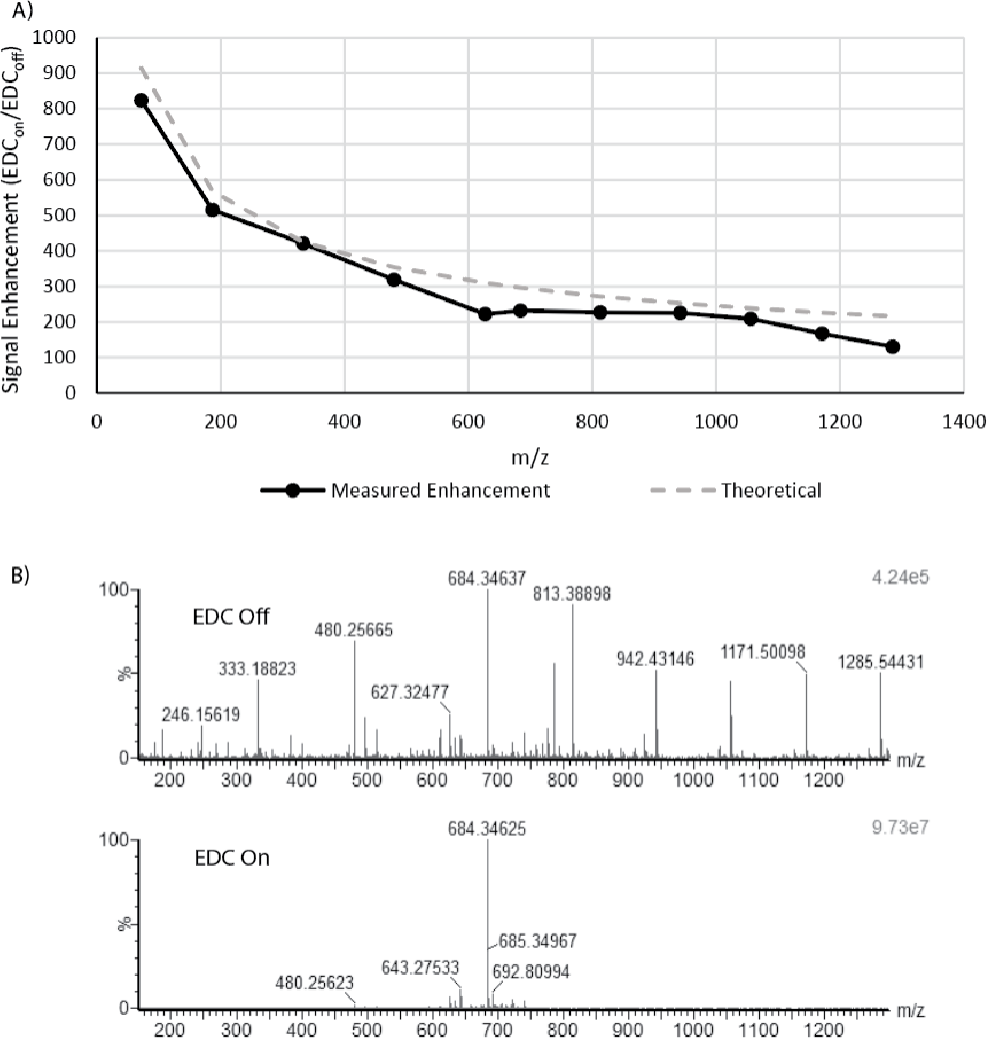
(A) Measured MSMS intensity factor increases for *N* = 1 analyzer passes observed with [Glu]–Fibrinopeptide B fragment ions when using EDC. The delay of the release of ions from the collision cell to the orthogonal accelerator (*T*_1_ – *T*_0_) was varied from 24 to 93 μs for *m*/*z* 72 and 1285, respectively, to maximize the duty cycle for a specific *m*/*z*. Data was acquired for a single constant push period of 4 ms, demonstrating on average 2 orders of magnitude signal increases. (B) Spectra showing relative signal increases for a targeted *m*/*z* of 684.

Due to the synchronous timing of ion arrival with the push within the orthogonal accelerator, a discrete range of *m*/*z* is transmitted through the ToF. This is advantageous for this application since *m*/*z* dependence of the ion arrival time within the orthogonal accelerator can be easily coupled with the time dependent nature of containing ions in the analyzer for multiple passes. Careful tuning of the pulse width and magnitude of the trapping potential in the collision cell can be used to maximize the duty cycle recovery. Increasing the pulse width can also increase the *m*/*z* range transmitted to the orthogonal accelerator. The enhancement in signal for a given *m*/*z* can be estimated from the reciprocal of the duty cycle over a fixed push period. Figure 3 demonstrates the signal increase observed for a range of single charged [Glu1]–Fibrinopeptide B fragment ions compared to EDC disabled with a 4 ms push period. As can be observed in Figure 3A, the signal increases are significantly higher for lower *m*/*z* ions due to their lower fill times *t*_fill_.

### ToF Aberrations after Multiple Passes

To increase resolving power for multiple passes, ToF aberrations Δ*t*_aber_ must not increase arrival time distributions 2Δ*t* at a faster rate than the increased arrival time *T*. For a single pass of the analyzer, the flight time *T* for *m*/*z* 1000 on the system described is approximately 1.3 ms with a path length of 47 m and an arrival time distribution of 2Δ*t* = 6 ns*.* The aberration coefficients contributing to Δ*t*, particularly for higher-order terms, are nonzero and accumulate over many passes. The most dominant effects were therefore investigated using SIMION 8.1^17^ to model the MRT analyzer and estimate limitations to increasing resolving power. Initial ion conditions were varied in *y*, *z*, and kinetic energy *E*_k_ in order to investigate variations in the ion arrival distribution. This was repeated for multiple analyzer passes to highlight the magnitude of aberrations generated in the analyzer.

**Figure 4:**
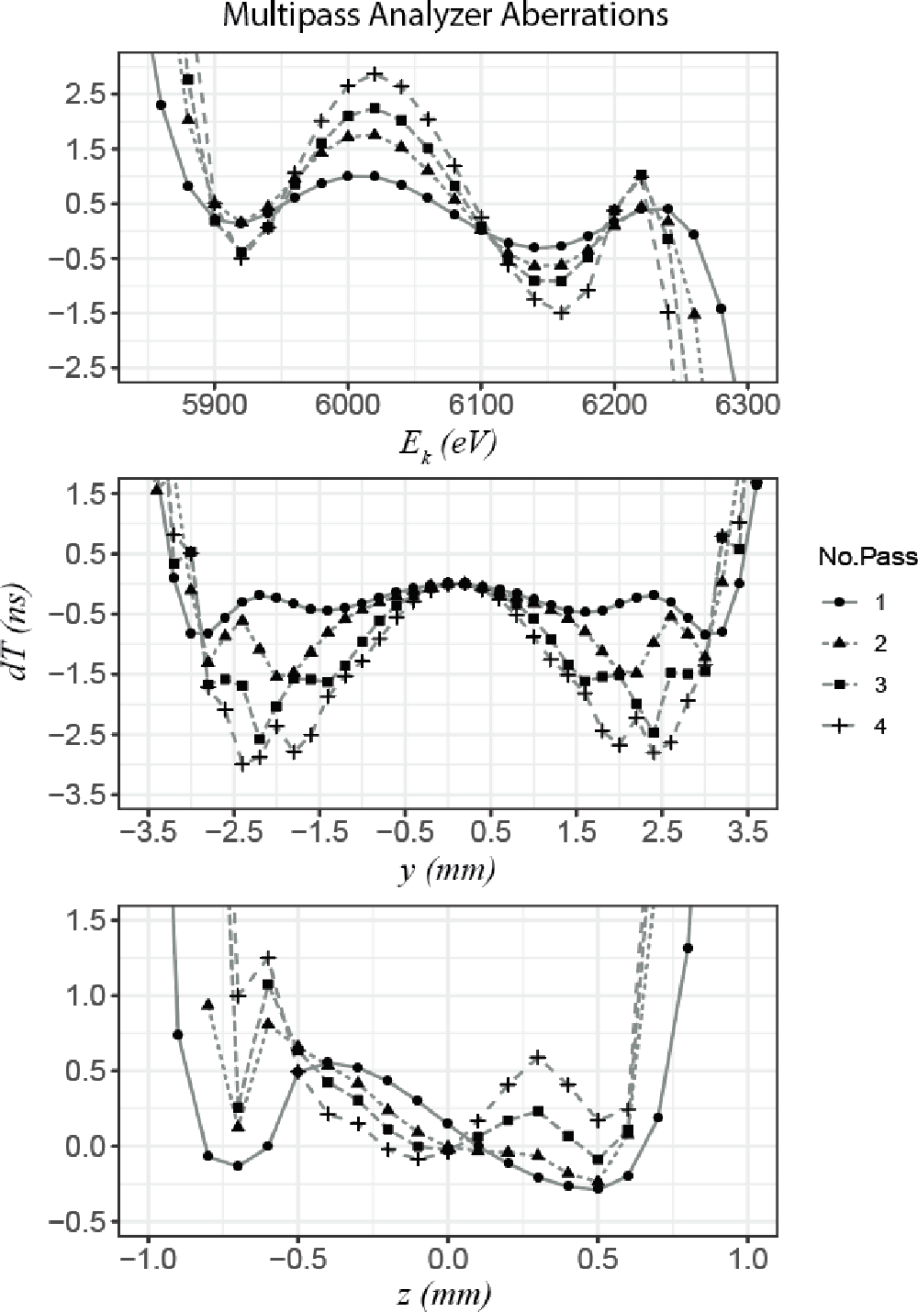
SIMION modeling of relative ion arrival time distribution d*T* (ns) for a range of initial ion conditions. Relative kinetic energies (*E*_k_) are varied from the reference energy of 6100 ± 300 eV. Single ion positions in the *y*- and *z*-axis are varied by ±4 and ±1 mm, respectively. Series are shown for one, two, three, and four analyzer passes, illustrating the change in arrival time distribution d*T*.

Figure 4 shows variation in ion arrival time due to beam width in the *y*-axis varies over 1 ns after one pass, while for four passes this extends out to 3 ns over 6 mm. The energy acceptance for one pass results in an ion arrival distribution over 1 ns, which is achieved for an energy range of 300 eV with a reference energy of 6100 eV. This highlights the effect of fourth-order isochronous focusing achieved on the multireflecting time-of-flight system. For four analyzer passes, the arrival time distribution increases to 4.5 ns for an equivalent energy range of 300 eV. For a beam width of 1 mm in the *z*-axis, the ion arrival time distribution remains below 1 ns over a 1 mm range for four analyzer passes. For *m*/*z* 1000 with a reference energy of 6100 eV, ion arrival time was modeled to be 1.37 and 5.3 ms for one and four passes, respectively.

SIMION modeling suggests ToF aberrations with respect to energy dispersion and beam height *y* have the largest impact on ion arrival times over multiple analyzer passes. Figure S5 demonstrates the effect of energy dispersion on ion arrival time after multiple analyzer passes for typical initial beam starting conditions in the orthogonal accelerator. ToF aberrations as a result of angular dispersion of the beam in the *z*-axis (α) and *y*-axis (β) were also investigated in Figure S6, with an α of <0.1° contributing to an arrival time distribution of 4 ns for one analyzer pass. Arrival time distribution did not increase significantly over multiple passes, suggesting careful beam conditioning prior to orthogonal acceleration can be maintained over multiple passes. Angular dispersion β contributed to 1 ns for one pass and up to 3 ns for four passes over 0.2°. The initial velocity distribution and beam conditioning prior to orthogonal acceleration are important factors in minimizing Δ*t*.

## Experimental Section

### Resolving Power and Ion Transmission over Multiple Passes

Resolving power improvements for multiple analyzer passes were assessed on a prototype MRT analyzer developed for research purposes. The analyzer dimensions are 100 × 45 cm, using *y*-injection whereby the height *y* of the beam was approximately 6 times greater than the *z*-width (∼6 × 1 mm). The analyzer pressure was 1.0 × 10^–8^ mbar. The push interval was varied from 4 to 5.5 ms to accommodate flight times for up to four passes of *m*/*z* 785 (5082 μs). Since ion accumulation with enhanced duty cycle is synchronous with the push frequency, the ion accumulation time within the collision cell was also extended to the corresponding push period, up to 5.5 ms. Ion accumulation periods greater than this were found to have a significant decrease in signal observed, suggesting limitations on the ion accumulation efficiency within the collision cell for greater than 5.5 ms. The *P1* pulse start time and durations were configured to the *m*/*z* value of interest. For *m*/*z* 556, *P1* start (*T*_2_) was set to 600 μs for *N* > 1 passes with pulse durations of 500, 1700, 2700, and 3600 μs for consecutive numbers of passes. The triggering of the acquisition was delayed from the push time *T*_0_ by several milliseconds, depending on the number of analyzer passes required. This can help overcome ADC bandwidth limitations for extended flight paths. The sampling frequency was 1 GS/s, and all multi pass data (*N* > 2) were acquired at 1 Hz on an Acqiris SA220 ADC instrument. Solutions of 100 fmol/μL [Glu1]–Fibrinopeptide B in 50:50 MeOH:H_2_O + 1% acetic acid, 10 ng/μL sulfadimethoxine in 1:1 MeCN:H_2_O + 0.1% formic acid, and 2 ng/μL leucine enkephalin in 50:50 ACN:H_2_O + 0.1% formic acid were sampled via electrospray at 5 μL/min to evaluate resolving power increases, duty cycle increases, and ion transmission for multiple analyzer passes.

### LC-MS Experiments for Two Analyzer Passes

To explore the impact of multiple analyzer passes upon small molecule identification, the pulse timings on *P1* were configured to allow two analyzer passes for an *m*/*z* range of 200 to 600, and the collision cell DC bias pulse width was extended to cover this *m*/*z* range at the expense of lower duty cycle enhancements achieved for lower *m*/*z*. LC-MS urinary screening of a healthy volunteer patient has been performed 4 h post dose of 1000 mg of acetaminophen. The donor sample was diluted 1:10 with water and injected (5 μL) onto an ACQUITY UPLC HSS T3 C18 (100 mm × 2.1 mm, 1.8 μm) column held at 40 °C fitted to a UHPLC interfaced to a SELECT SERIES MRT mass spectrometer via an electrospray source. The sample was separated over a 12 min gradient of water + 0.1% formic acid (MPA) and acetonitrile + 0.1% formic acid (MPB) (starting composition 0.5 mL/min 99% MPA held for 1 min, ramping to 85% MPA over 2 min, ramping to 50% MPA over 3 min, and ramping to 5% MPA over 3 min, which is held for 1 min, then returning to 99% MPA over 0.1 min and reconditioning for 1.9 min at 99% MPA). Data were acquired between 200 and 600 *m*/*z* utilizing two passes of the analyzer at an acquisition rate of 10 Hz. Data were processed using MassLynx version 4.2 software and were lockmass corrected using leucine enkephalin ([M + H]^+^, *m*/*z* 556.27658) introduced via lockspray.

### DESI Experiments for Two Analyzer Passes

A healthy wild-type mouse kidney was axially cryosectioned onto standard glass slides at a thickness of 16 μm. The sections were analyzed on a DESI XS source fitted to an MRT mass spectrometer in duplicate, employing either a single or double pass of the analyzer. DESI spray conditions were set at 2 μL/min, 95:5 MeOH:water with 100 pg/μL Leu–enkephalin with 0.86 kV and 15 psi nebulizing gas applied to the DESI high performance sprayer,^18^ and the heated transfer line was maintained at 350 °C. Acquisitions were performed in full scan MS at 2 Hz in positive ionization mode. Data were processed using High Definition Imaging (HDI version 1.7) software to generate ion images for highlighting the different regions of the kidney (cortex and medulla).

## Results

### Resolving Power and Ion Transmission over Multiple Passes

**Figure 5:**
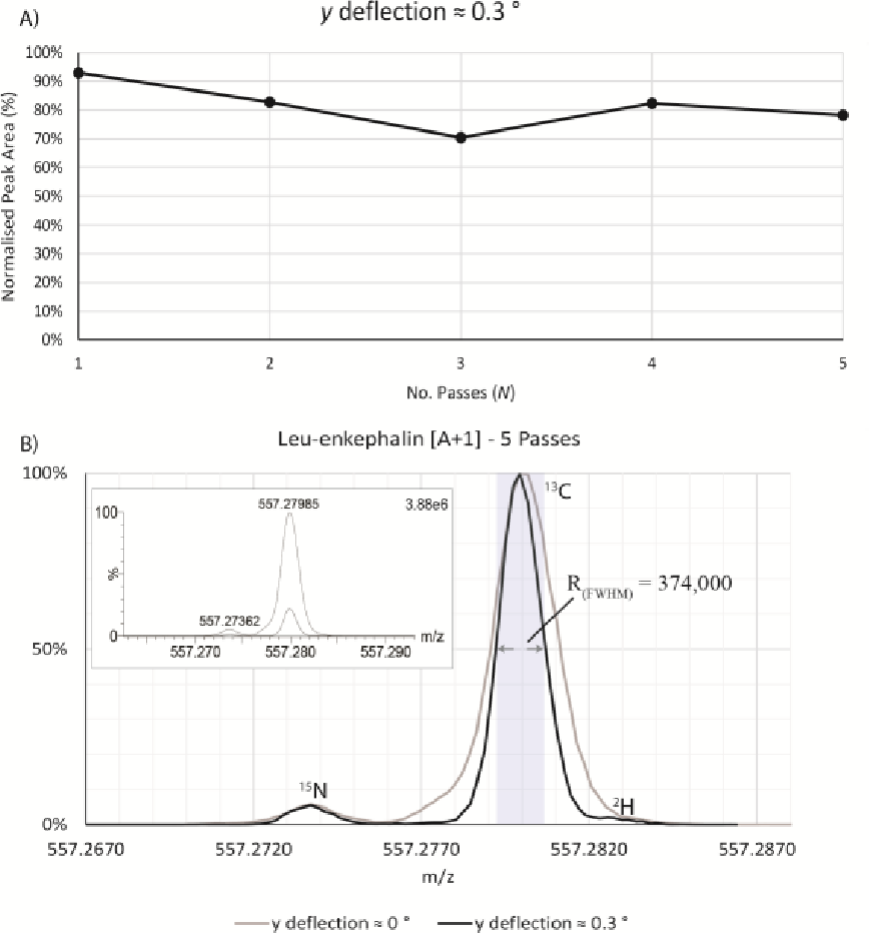
(A) Approximately 80% beam transmission after five analyzer passes was measured on leucine enkephalin [A+1] at *m*/*z* 557 with *y*-deflection ∼0.3° to maximize resolution. Data were averaged over three repeated experiments with peak area normalized to the first analyzer pass. (B) Comparison of leucine enkephalin [A+1] spectra after five analyzer passes; varying the *y*-deflection led to increases in resolving power from 269,000 to 374,000 (fwhm), suggesting the *y*-aberrations may have limited resolution on this system. Signal intensities have been normalized to illustrate the resolution improvements, while the inset shows overlaid spectra with relative intensities.

Resolving power and ion signal were monitored to assess the trapping efficiency of the analyzer for *N* multiple passes. Initial observations after five analyzer passes showed a lower than expected resolving power of 269,000 (Figure 5B).

The data in Figure 4 demonstrate that aberrations contributed to by the *y*-profile and energy dispersion of the beam had a significant impact on ion arrival time, particularly for multiple analyzer passes.

The beam profile in the *y-*axis was therefore investigated further to assess whether resolving power improvements could be obtained. The *y*-deflection of the beam after the orthogonal accelerator was adjusted, leading to an approximate 0.3° deflection in the *y*-axis, resulting in a smaller proportion of the beam transmitted through the analyzer; simulations suggest the resulting ion transmission to be approximately 10% after five analyzer passes, with the majority of losses occurring during the first reflections due to collisions with the *y*-aperture at the entrance to the mirrors. This led to an increase in the resolving power to 374,000 (fwhm) at *m*/*z* 557.28 (Figure 5B), suggesting the effect of the *y* and *E*_k_ aberrations on the ion arrival time distribution may limit achievable resolving power.

With the beam deflected in the *y*-axis, the *m*/*z* peak areas were monitored to assess ion transmission for *N* multiple analyzer passes. Ion transmission of 70% to 80% was observed over five analyzer passes (Figure 5A), suggesting a high trapping efficiency of the analyzer. The relative intensity was approximately 15% after *y*-deflection was introduced (Figure 5 B, inset). A large proportion of this signal loss is on the first entrance to the analyzer and a result of initial collisions with the 6 mm entrance aperture on the mirror assembly. Since the analyzer is shown to have minimal losses for multiple passes, phase space improvements within the orthogonal accelerator may lead to increases in resolving power without a reduction in ion transmission.

**Figure 6:**
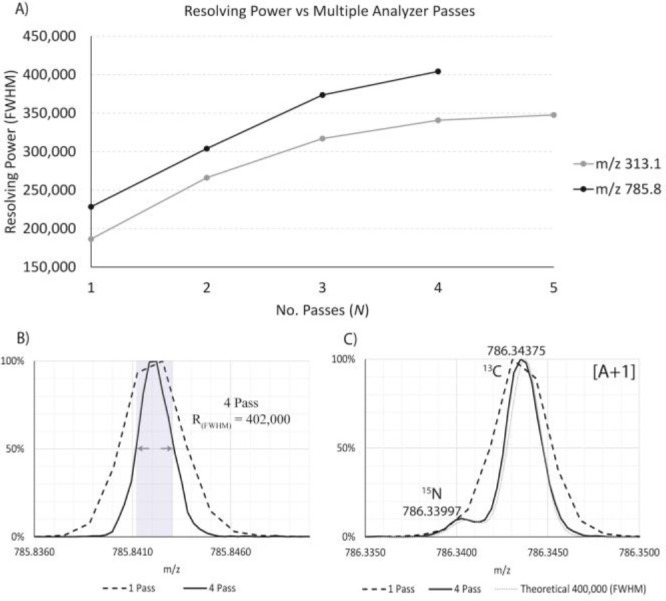
(A) Measured resolving power (fwhm) increases for multiple analyzer passes for *m*/*z* 313.1 (sulfadimethoxine [A+2]) and *m*/*z* 785.8 ([Glu1]–Fibrinopeptide B [M + 2H]^2+^). After four passes, resolving power increased to 402,000 (fwhm) for *m*/*z* 785 while 344,000 (fwhm) was achieved for *m*/*z* 313.1 at five analyzer passes. Resolving power increases were observed to diminish after four passes. (B) Comparison in resolving power for [Glu1]–Fibrinopeptide B [M + 2H]^2+^ after one and four passes. (C) [Glu1]–Fibrinopeptide B [A+1] after four passes illustrates ^15^N beginning to be resolved from ^13^C. Dotted series shows the theoretical fine isotope structure for the base peak resolving power of 400,000 (fwhm).^19^

Increases in resolving power observed at multiple analyzer passes for a range of *m*/*z* were demonstrated. Resolving power at *m*/*z* 785.84 increased from 222,000 for one pass to 402,000 (fwhm) at four passes (Figure 6B). Comparative spectra demonstrating measured resolving power of 187,000 (fwhm) and 344,000 (fwhm) were measured for one and five passes, respectively, for *m*/*z* 313.1 in Figure S2. After four passes, the resolution increases were broadly observed to remain static, suggesting aberrations within the analyzer were contributing significantly to the arrival time distributions.

Additional data were also gathered at a range of scan rates for *m*/*z* 785.8, including 1, 10, and 30 Hz shown in Figure S3. For *N = 2* analyzer passes, the resolution is maintained at approximately 320,000 (fwhm).

**Figure 7:**
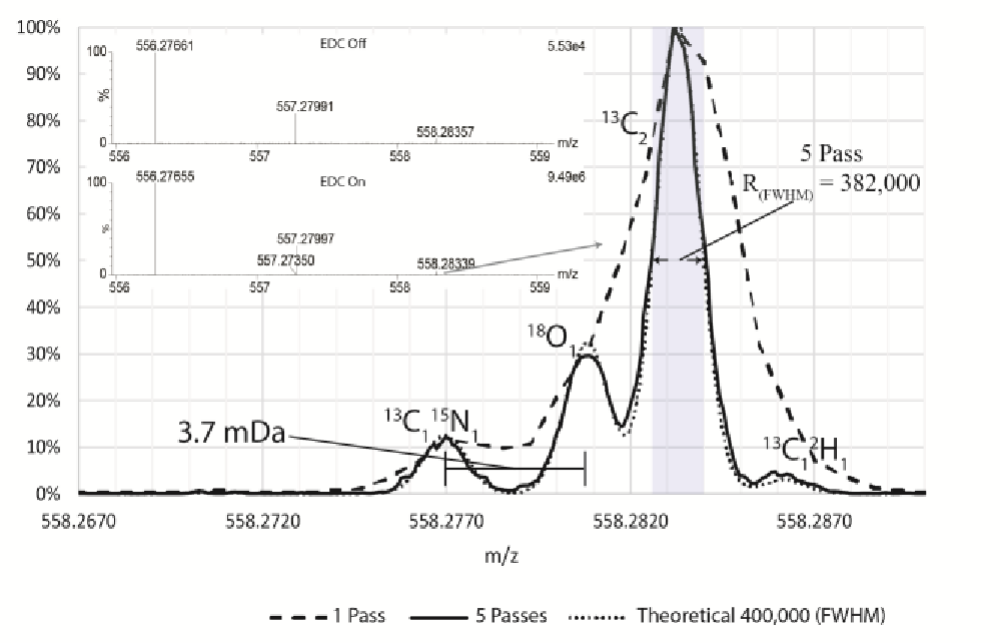
Comparison of the Leu–enkephalin [A+2] isotope after five analyzer passes with theoretical fine isotope structure at 400,000 (fwhm) shown in the dotted series. The base peak resolving power (fwhm) was measured at 382,000, which corresponds to an arrival time *T* of 4654.8 μs and 2Δ*t* = 12.2 ns. Dashed series shows comparative spectra for a single analyzer pass. Inset shows zoomed out spectra with and without EDC, demonstrating a signal increase of 2 orders of magnitude.

Figure 7 highlights the benefits of higher resolving power, yielding the fine isotope structure of Leu–enkephalin [A+2]. The comparison at 200,000 (fwhm) resolving power (one analyzer pass) is also shown, highlighting the additional information revealed for *N* = 5 analyzer passes. The change in signal recovery without duty cycle enhancement (inset) showed an increase of 2 orders of magnitude.

### Space Charge Effects

**Figure 8:**
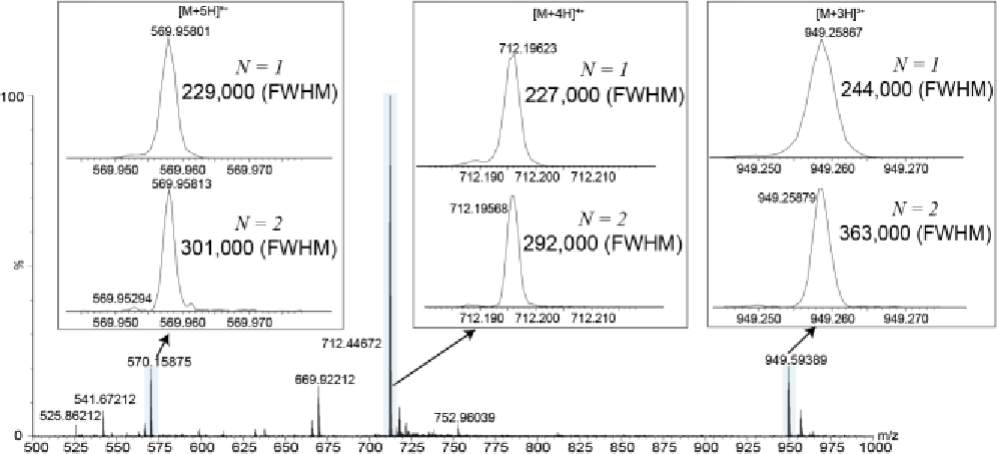
Melittin example showing resolution changes at +3, +4, and +5 charge states for one or two analyzer passes. Higher resolving power increases were observed for lower charge states.

For high ion signals and particularly high charge states, reductions in resolving power were observed. Melittin was acquired to assess changes in resolution for varying charge states, illustrating observed resolving power decreases for higher charge states Figure 8. For *N* = 1 analyzer pass, the change in resolution observed is minimal; however, for *N* = 2 analyzer passes, the increase in resolving power is lower for higher charge states. In general, for ion signals of >20 charges per push for a given *m*/*z*, peak broadening and subsequent resolving power decreases were observed. An example is given for the monoisotopic Leu–enkephalin peak at approximately 17 charges per push and 55 charges per push with a reduction in resolving power of 305,000 (fwhm) to 249,000 (fwhm), respectively, in Figure S6.

### LC-MS: Two Analyzer Passes

**Figure 9:**
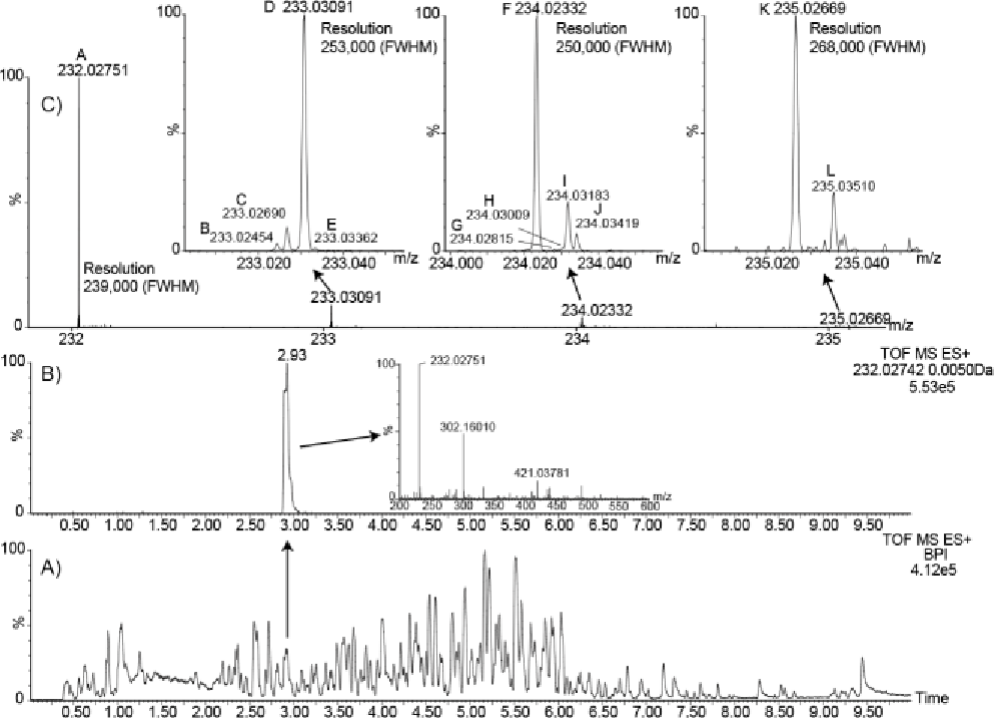
Data from two analyzer passes for an LC-MS metabolite identification acquisition of a urine sample at 10 Hz. (A) BPI chromatogram of 4 h post dose urine sample. (B) Extracted ion chromatogram of *m*/*z* 232.02742, where the inset is the mass spectrum of the peak at 2.93 min showing base peak of *m*/*z* 232.02751, which corresponds to the [M + H]^+^ of acetaminophen sulfate (C_8_H_9_NO_5_S). (C) Mass spectrum showing the isotopic envelope of acetaminophen sulfate, where the inset depicts expanded regions showing the fine isotope structure (label annotation is shown in Table S1). Note, all mass spectra are annotated with the centroid accurate mass-to-charge ratio.

The benefits of utilizing two analyzer passes can be seen in Figure 9, when performing LC-MS (10 Hz) at >300,000 (fwhm), Figure 9A shows the BPI for the LC-MS analysis and Figure 9B shows the extracted ion chromatogram for acetaminophen sulfate (232.02742 *m*/*z*, C_8_H_9_NO_5_S). A total of 12 peaks were resolved for the combined isotopic distribution with four isotopes observed for the A+1, five isotopes for the A+2, and three isotopes for the A+3 fine isotope distributions (insets in Figure 9C). The observed mass accuracies of these isotopes are summarized in Table S1, where the overall observed mass measurement accuracy was 603 ppb RMS for the 12 observed isotopes of acetaminophen sulfate.

Comparative data at scan rates of 10 and 30 Hz for an LC-MS analysis are shown in Figure S4. Sulfadimethoxine fine isotope structure for the A+2 is shown in detail. Resolving power of the base peak at *m*/*z* 313.1 was measured at 254,000 (fwhm) at both scan rates.

The combination of sub ppm mass measurement accuracy and the ability to resolve fine isotope structure and confirm putative empirical formulas significantly increases confidence in identification of both known and unknown species within complex samples.

### DESI: Two Analyzer Passes

**Figure 10:**
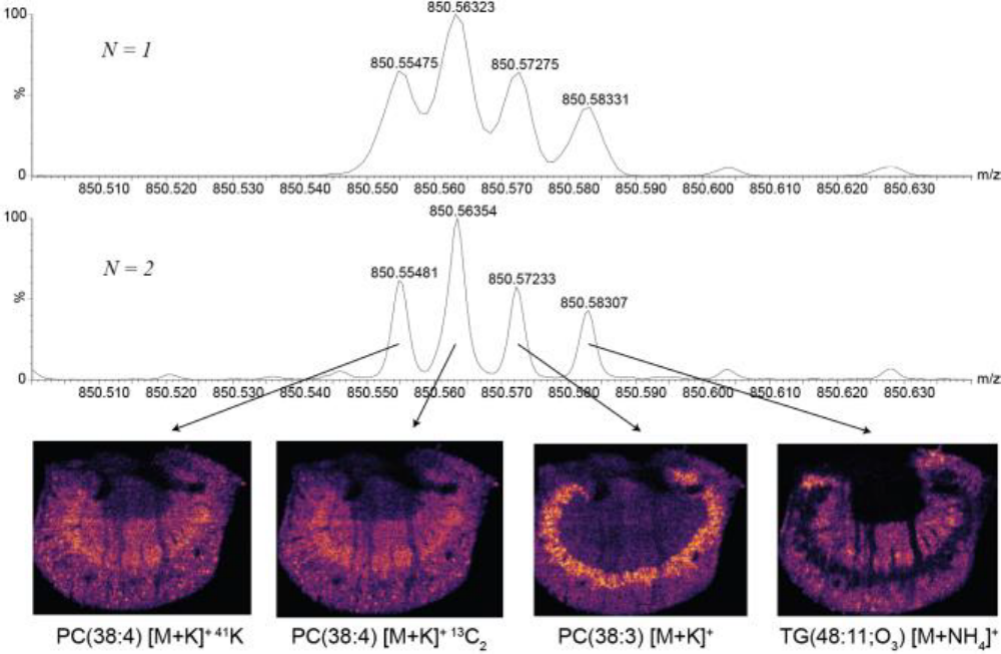
Single vs two passes of the analyzer mass spectrometry imaging spectra acquired from the mouse kidney tissue section at *m*/*z* 850.56 showing MS resolution improvement at >300,000 fwhm with baseline peak separation resulting in improved imaging specificity. Putative identifications were obtained from the LipidMaps database.

Figure 10 demonstrates the improvement observed with increased *m*/*z* resolving power with a baseline resolution of four peaks within a 30 mDa window centered around *m*/*z* 850.56. Putative identification using the LIPID MAPS^20^ database provided two isotopic peaks from the fine isotope structure ^13^C and ^41^K of the lipid PC(38:4), generating the same ion images. The other peaks were identified as potassiated PC(38:3) and an ammonium adduct of TG(48:11;O3).

## Conclusion

Multiple passes within a multireflecting ToF analyzer resulted in resolving powers of greater than 400,000 (fwhm) at *m*/*z* 785. High-order ToF focusing was demonstrated experimentally and via simulation to achieve increasing resolving power for up to four analyzer passes, after which flight time aberrations with respect to ion beam conditions, such as beam width (*y*), angular dispersion in the *y*-axis, and energy dispersion (*E*_k_), were found to limit resolving power. Introducing a small angular deflection in *y* increased the measured resolving power, suggesting that *y*-aberrations may have been a limiting factor on the system described. The arrival time distributions are relatively large for five analyzer passes (*m*/*z* 558, 2Δ*t* = 12.2 ns), suggesting future improvements to further reduce higher-order aberrations may lead to significant increases in resolving power. The coupling of a method to increase duty cycle with multiple analyzer passes demonstrated signal increases of 2 orders of magnitude, with the greatest increases observed for lower *m*/*z*. The combination of multiple analyzer passes with enhanced duty cycle has enabled high resolving powers at fast scan speeds (30 Hz), enabling the analysis of fine isotope structure. The approach has been applied to MS imaging and fast separation techniques, demonstrating significant potential for improvements in analyte identification for a wide range of applications.

## Supporting Information Available

Fine isotope structure of marbofloxacin after *N* = 2 analyzer passes and sulfadimethoxine after *N* = 5 analyzer passes, comparison of resolution for 1, 20, and 30 Hz, simulation data showing relative energy spread and ion arrival time distributions for multiple analyzer passes, example spectra demonstrating space charge effects on resolution for 55 charges per push, and detailed mass measurement accuracy results in relation to Figure 9 (PDF)
