# Supplementary material for: Combining Enhanced Resolving Power with Duty Cycle Improvements on a Multi-Reflecting Time-of-Flight Mass Spectrometer
Source: J Am Soc Mass Spectrom. 2024 Aug 9;35(9):2073–81. doi: 10.1021/jasms.4c00122 (PMC11378273; doi:10.1021/jasms.4c00122)
Supplement: Supplementary file 1 — js4c00122_si_001.pdf [file js4c00122_si_001.pdf]

Supporting Information

Combining Enhanced Resolving Power with Duty Cycle Improvements on a Multi-Reflecting Time-of-Flight Mass Spectrometer

William J. Johnson[1*], Martin E. Palmer[1], Emmanuelle Claude[1], Michael McCullagh[1], Peter Nixon[1], Jason Wildgoose[1]

1. Waters Corporation, Stamford Avenue, Altrincham Road, Wilmslow, Cheshire, UK, SK9 4AX
*William Johnson email: william_johnson@waters.com

Figure S1 - Marbofloxacin acquired with ESI direct infusion at 5 µL/min with a 2 Hz scan rate................ 2
Figure S2 - Comparison of Sulfadimethoxine spectra displayed for one analyzer pass (Red) and five analyzer passes (Green). 2
Figure S3 – Comparison of 100 fmol [Glu[1]]-Fibrinopeptide B via direct infusion at 5 µl/min, acquired at 1, 10, and 30 Hz for N = 2 analyzer passes.. 3
Figure S4 – LCMS experiment comparison at 10 and 30 Hz for N = 2 analyzer passes demonstrating no significant change in resolution. 4
Figure S5 – Simulation of 500 ions of m/z 1000 after N multiple passes of the MRT analyzer. 5
Figure S6 – Arrival time distributions for multiple analyzer passes with varying angular dispersion in the z-axis (α) and y-axis (β)... 6
Figure S7 - Example of Leu-Enkephalin M-H, at 17 charges per push per m/z with expected resolution after N = 2 analyser passes (Top). 7

Table S1 - Summary of observed mass accuracy for the fine isotopes of acetaminophen sulfate shown in Figure 9. 4

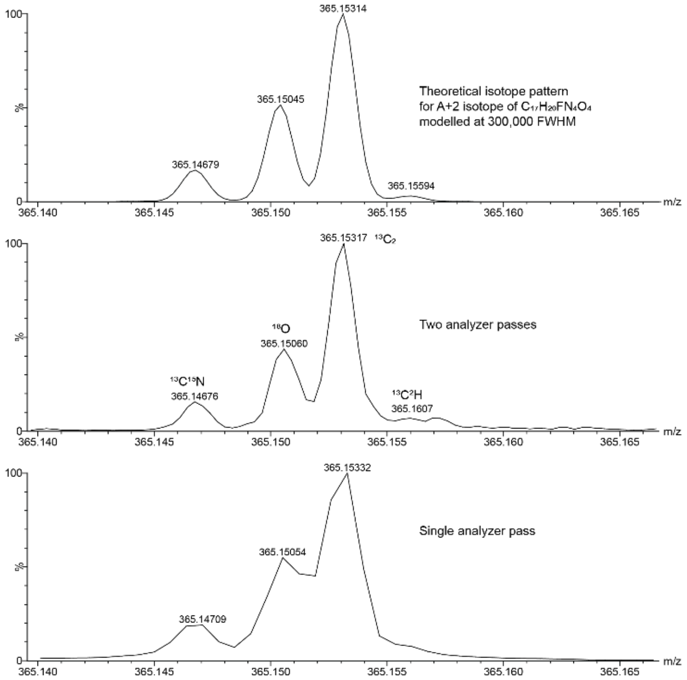

Figure S1 – Marbofloxacin [A+2] acquired with ESI direct infusion at 5 µL/min with a 2 Hz scan rate. Resolution increases to >300,000 after N = 2 analyzer passes and fine isotope structure $^{13}C^{15}N$, ^{18}O, ^{13}C, $^{13}C^{2}H$ begins to be revealed.

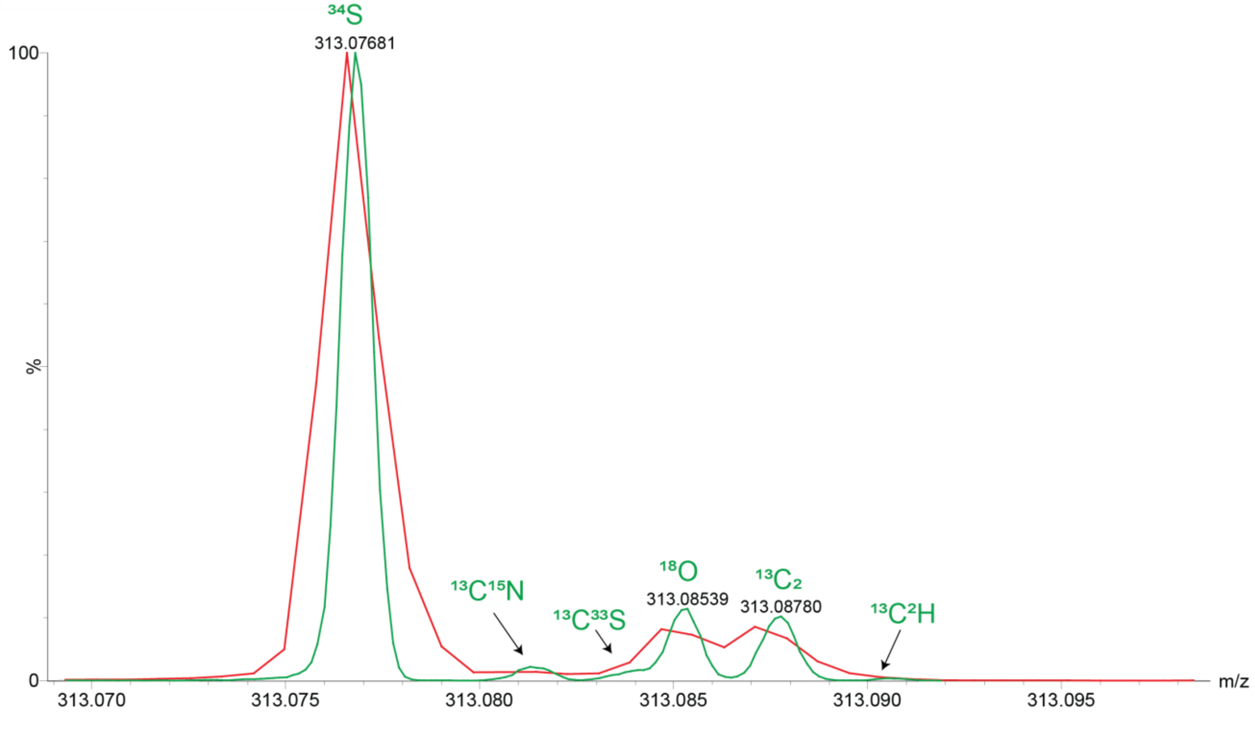

Figure S2 - Comparison of Sulfadimethoxine spectra displayed for one analyzer pass (Red) and five analyzer passes (Green). The base peak resolution (FWHM) at m/z 313 for 1 pass is measured at 187,422 whilst N=5 passes is 344,219.

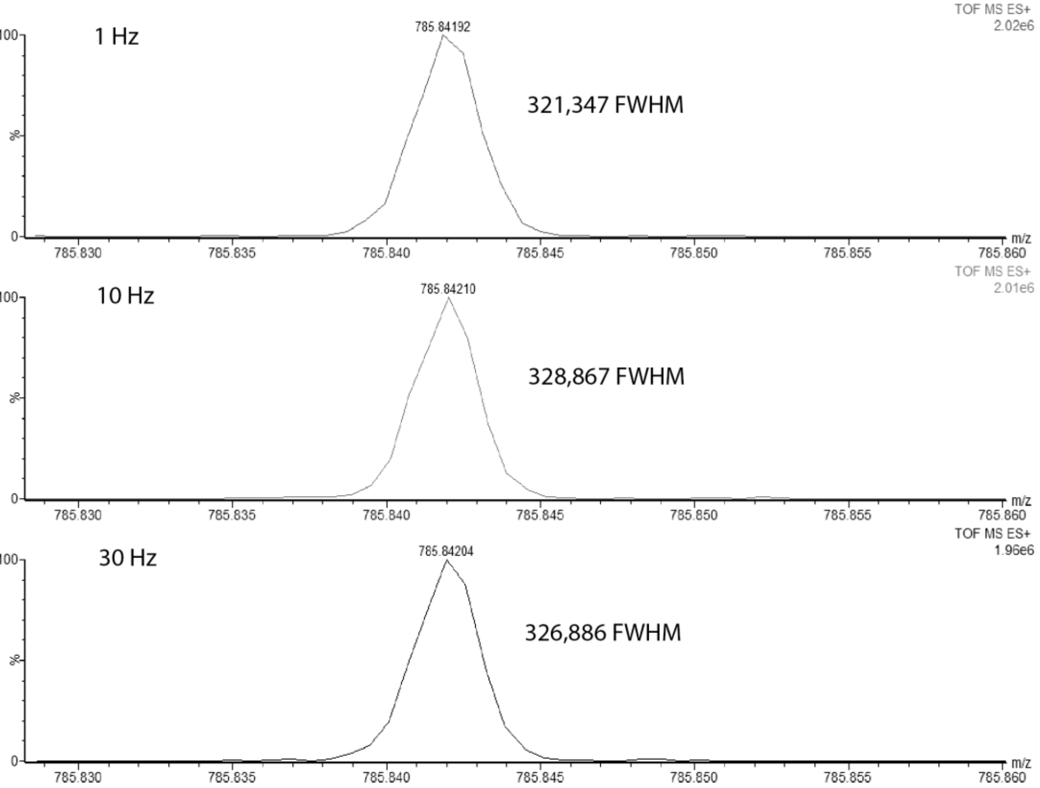

Figure S3 – Comparison of 100 fmol [Glu¹]-Fibrinopeptide B via ESI direct infusion at 5 µl/min, acquired at 1, 10, and 30 Hz for N = 2 analyzer passes.

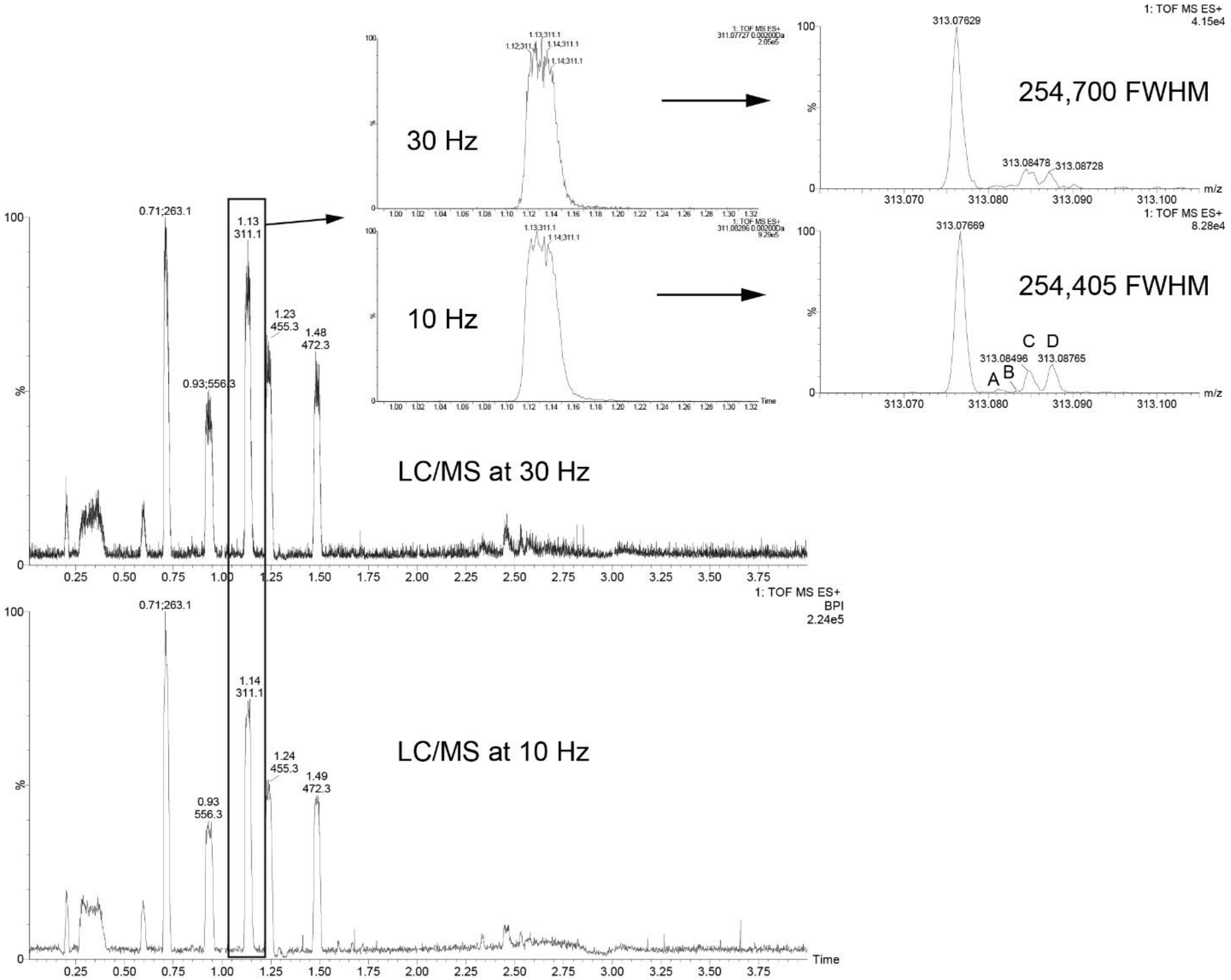

Figure S4 – LC-MS experiment comparison at 10 and 30 Hz for N = 2 analyzer passes demonstrating no significant change in resolving power for Sulfadimethoxine [A+2] at m/z 313.08. Fine isotope structure is beginning to be resolved for A) $^{13}C_1$ $^{15}N_1$, B) $^{13}C_1{}^{33}S_1$, C) $^{18}O_1$, and D) $^{13}C_2$.

Empirical Formula	Adduct	Label	Expected *m/z*	Observed *m/z*	mDa error	ppb error
$^{12}C_8{}^1H_9{}^{14}N_1{}^{16}O_5{}^{32}S_1$	H^+	A	232.02742	232.02751	0.09	388
$^{12}C_8{}^1H_9{}^{15}N_1{}^{16}O_5{}^{32}S_1$	H^+	B	233.02445	233.02454	0.09	386
$^{12}C_8{}^1H_9{}^{14}N_1{}^{16}O_5{}^{33}S_1$	H^+	C	233.02681	233.02690	0.09	386
$^{12}C_7{}^{13}C_1{}^1H_9{}^{14}N_1{}^{16}O_5{}^{32}S_1$	H^+	D	233.03077	233.03091	0.14	601
$^{12}C_8{}^1H_8{}^2H_1{}^{14}N_1{}^{16}O_5{}^{32}S_1$	H^+	E	233.03370	233.03362	-0.08	-343
$^{12}C_8{}^1H_9{}^{14}N_1{}^{16}O_5{}^{34}S_1$	H^+	F	234.02322	234.02332	0.1	427
$^{12}C_7{}^{13}C_1{}^1H_9{}^{15}N_1{}^{16}O_5{}^{32}S_1$	H^+	G	234.02781	234.02815	0.34	1453
$^{12}C_7{}^{13}C_1{}^1H_9{}^{14}N_1{}^{16}O_5{}^{33}S_1$	H^+	H	234.03016	234.03009	-0.07	-299
$^{12}C_8{}^1H_9{}^{14}N_1{}^{16}O_4{}^{18}O_1{}^{32}S_1$	H^+	I	234.03167	234.03183	0.16	684
$^{12}C_6{}^{13}C_2{}^1H_9{}^{14}N_1{}^{16}O_5{}^{32}S_1$	H^+	J	234.03413	234.03419	0.06	256
$^{12}C_7{}^{13}C_1{}^1H_9{}^{14}N_1{}^{16}O_5{}^{34}S_1$	H^+	K	235.02657	235.02669	0.12	511

$^{12}C_7{}^{13}C_1{}^{1}H_9{}^{14}N_1{}^{18}O_5{}^{32}S_1$	H^+	L	235.03502	235.03510	0.08	340
				RMS error	0.15	603

Table S1 - Summary of observed mass accuracy for the fine isotopes of acetaminophen sulfate shown in Figure 9.

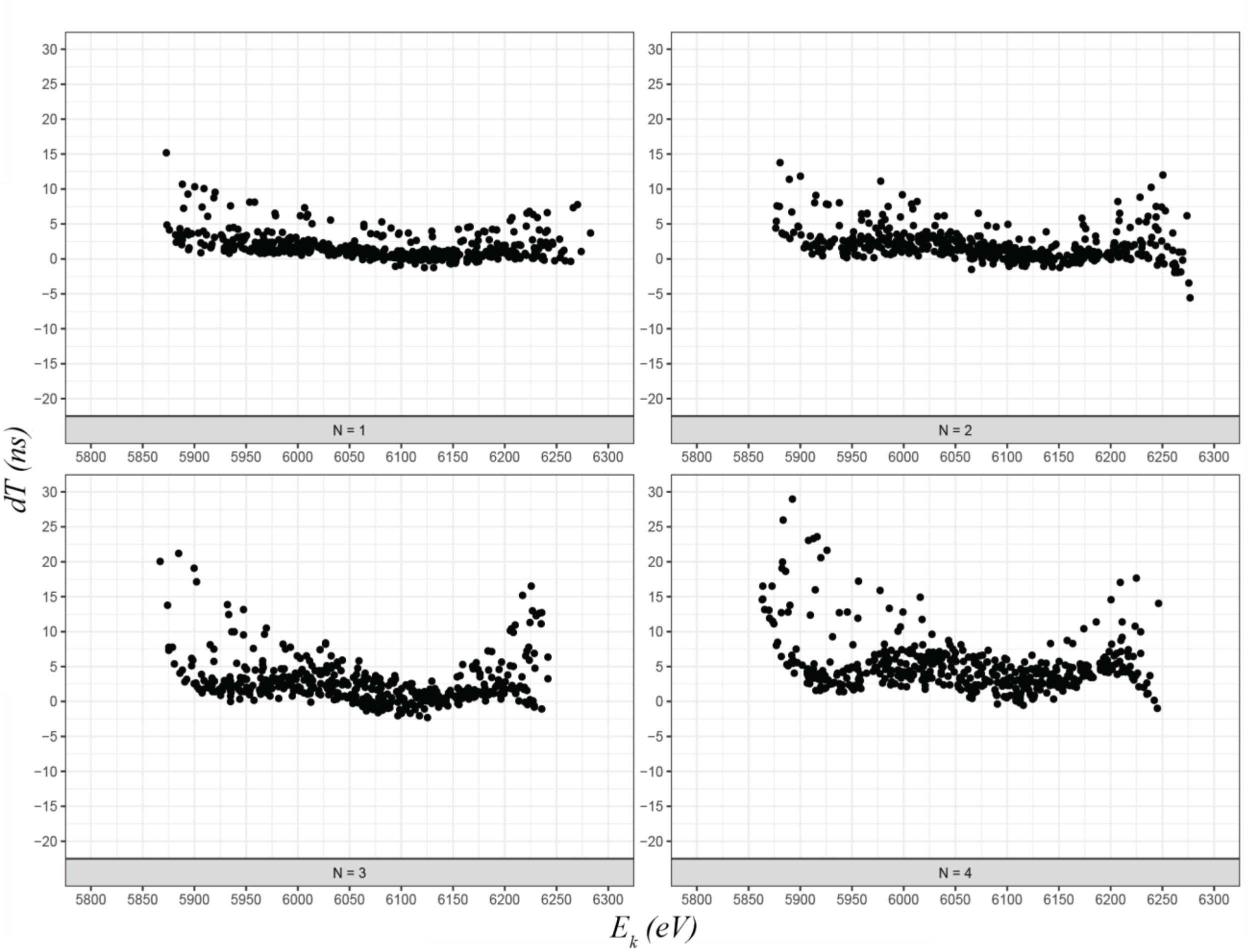

Figure S5 – Simulation of 500 ions of m/z 1000 after N multiple passes of the MRT analyzer. Initial beam conditions y = 8 mm, z = 1.0 mm, cone distribution of half angle 0.3 °, and 66 eV gaussian distribution 2 eV (FWHM). Ions are generated within the orthogonal accelerator along the axis of the push plane and off axis of the detector plane by 3° to account for energy of the beam in the y-axis. A subsequent two stage deflector allows steering of the beam into the multi-reflecting analyzer in both the y and z-axis. Plots demonstrate the dependence of energy dispersion on relative ion arrival time distribution (dT).

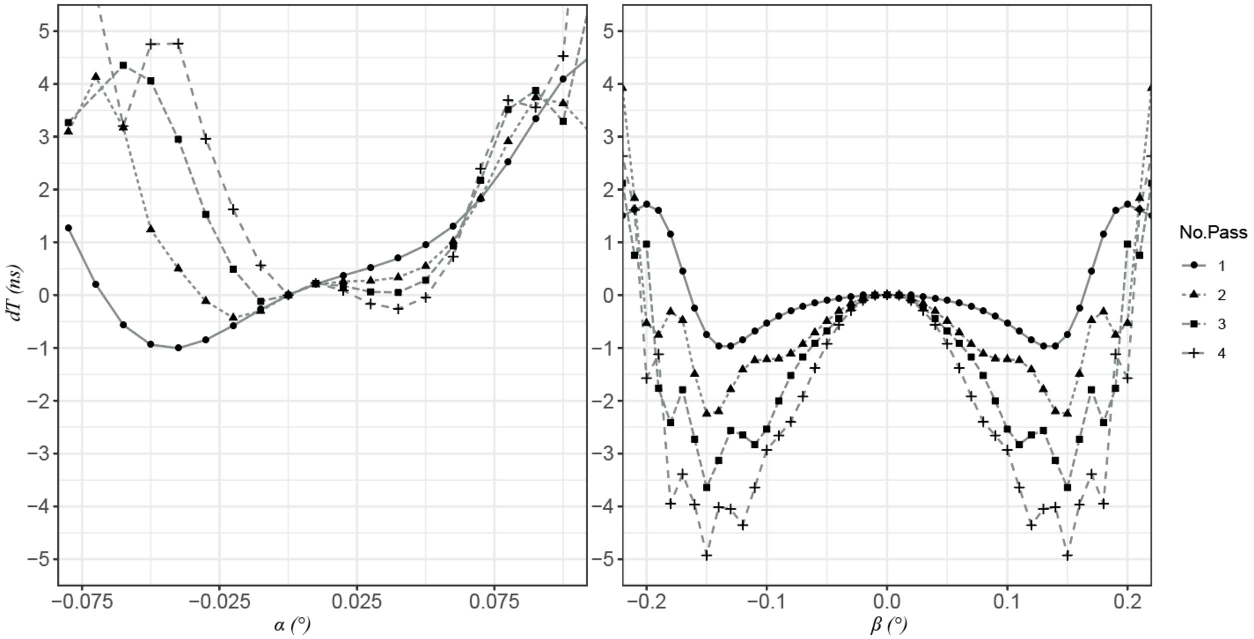

Figure S6 – Simulated arrival time distributions for multiple analyzer passes with varying angular dispersion in the z-axis (α) and y-axis (β).

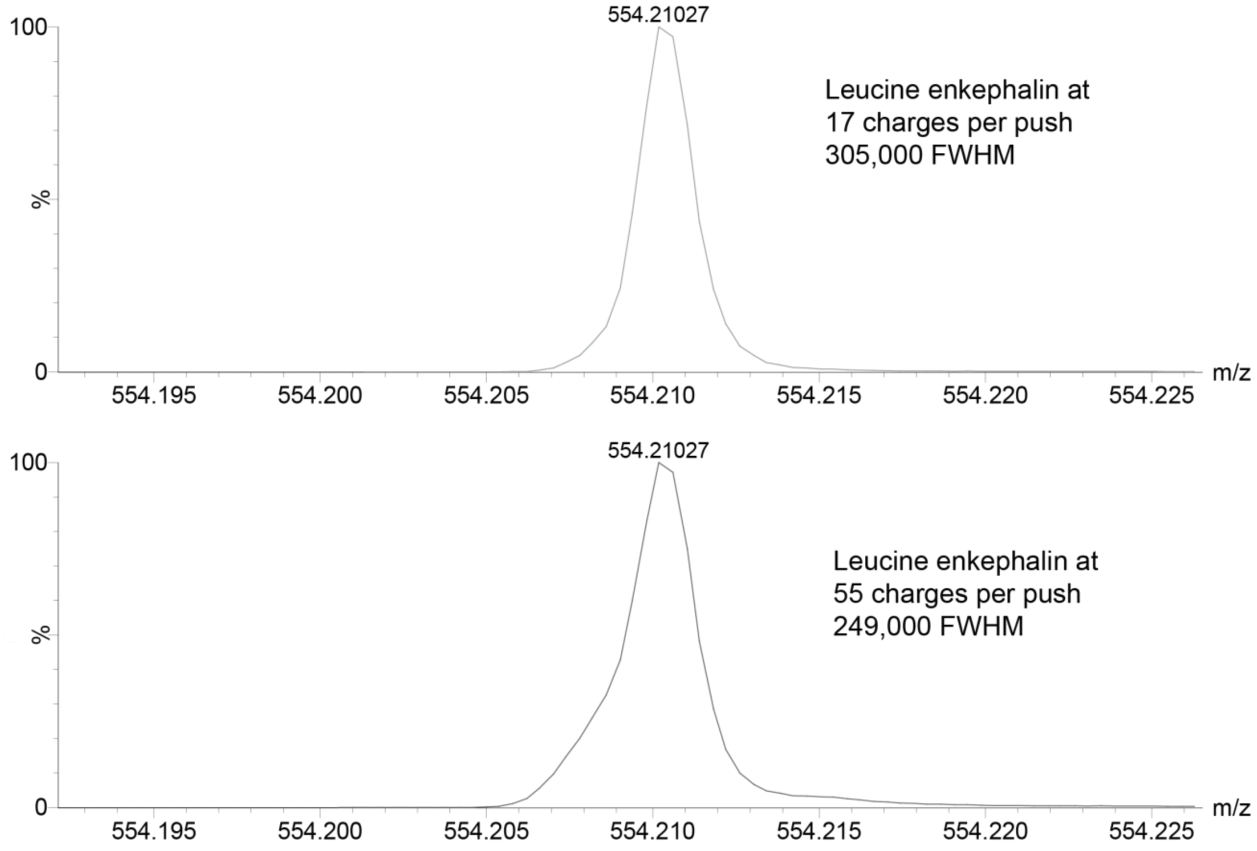

Figure S7 - Example of Leu-Enkephalin [M-H]$^{-}$, at 17 charges per push per m/z with expected resolving power after N = 2 analyser passes (Top). Whilst for 55 charges per push (Bottom) peak shape begins to be distorted and resolving power reduced to 249,000 (FWHM).
